# Supplementary material for: Formation of Abiogenic Hydrocarbons in Supercritical Fluids under Earth’s Upper Mantle Conditions
Source: JACS Au. 2026 Feb 25;6(3):1685–92. doi: 10.1021/jacsau.5c01558 (PMC13014190; doi:10.1021/jacsau.5c01558)
Supplement: Supplementary file 1 [file au5c01558_si_001.pdf]

Supporting information for:

Formation of abiogenic hydrocarbons in supercritical fluids under Earth's upper mantle conditions

Nore Stolte,[1] Tao Li,[1] and Ding Pan[1,2]

[1]*Department of Physics, Hong Kong University of Science and Technology, Hong Kong, China*

[2]*Department of Chemistry, Hong Kong University of Science and Technology, Hong Kong, China*

TABLE SI. The NVT simulations performed for mixtures of CO, H_2 and H_2O. Pressure was computed for the unreacted mixtures. The column labeled "Time" shows the total length of the simulation. Uncertainties are standard deviations.

Run	Starting composition	T (K)	P (GPa)	V (cm^3/mol)	Time (ps)
1	32 CO + 32 H_2	1400	13.3 ± 0.6	11.42	89.5
2	32 CO + 32 H_2	1400	13.3 ± 0.6	11.42	79.8
3	32 CO + 32 H_2	1400	13.3 ± 0.6	11.42	75.0
4	32 CO + 32 H_2	1400	13.3 ± 0.6	11.42	101.6
5	32 CO + 32 H_2	1400	13.3 ± 0.6	11.42	96.8
6	32 CO + 32 H_2	1400	13.3 ± 0.6	11.42	106.4
1	18 CO + 18 H_2 + 18 H_2O	1400	13.2 ± 1.0	11.44	79.8
2	18 CO + 18 H_2 + 18 H_2O	1400	13.2 ± 1.0	11.44	75.0
3	18 CO + 18 H_2 + 18 H_2O	1400	13.2 ± 1.0	11.44	79.8
4	18 CO + 18 H_2 + 18 H_2O	1400	13.2 ± 1.0	11.44	159.6
5	18 CO + 18 H_2 + 18 H_2O	1400	13.2 ± 1.0	11.44	56.3
6	18 CO + 18 H_2 + 18 H_2O	1400	13.2 ± 1.0	11.44	70.1
1	18 CO + 18 H_2 + 18 H_2O	1400	10.0 ± 0.9	12.72	205.6
2	18 CO + 18 H_2 + 18 H_2O	1400	10.0 ± 0.9	12.72	70.1
3	18 CO + 18 H_2 + 18 H_2O	1400	10.0 ± 0.9	12.72	125.8
4	18 CO + 18 H_2 + 18 H_2O	1400	10.0 ± 0.9	12.72	140.3
5	18 CO + 18 H_2 + 18 H_2O	1400	10.0 ± 0.9	12.72	75.0
6	18 CO + 18 H_2 + 18 H_2O	1400	10.0 ± 0.9	12.72	150.0
1	18 CO + 18 H_2 + 18 H_2O	1000	9.8 ± 0.9	12.16	60.5
2	18 CO + 18 H_2 + 18 H_2O	1000	9.8 ± 0.9	12.16	96.8
3	18 CO + 18 H_2 + 18 H_2O	1000	9.8 ± 0.9	12.16	89.5
4	18 CO + 18 H_2 + 18 H_2O	1000	9.8 ± 0.9	12.16	130.6
5	18 CO + 18 H_2 + 18 H_2O	1000	9.8 ± 0.9	12.16	116.1
6	18 CO + 18 H_2 + 18 H_2O	1000	9.8 ± 0.9	12.16	65.3

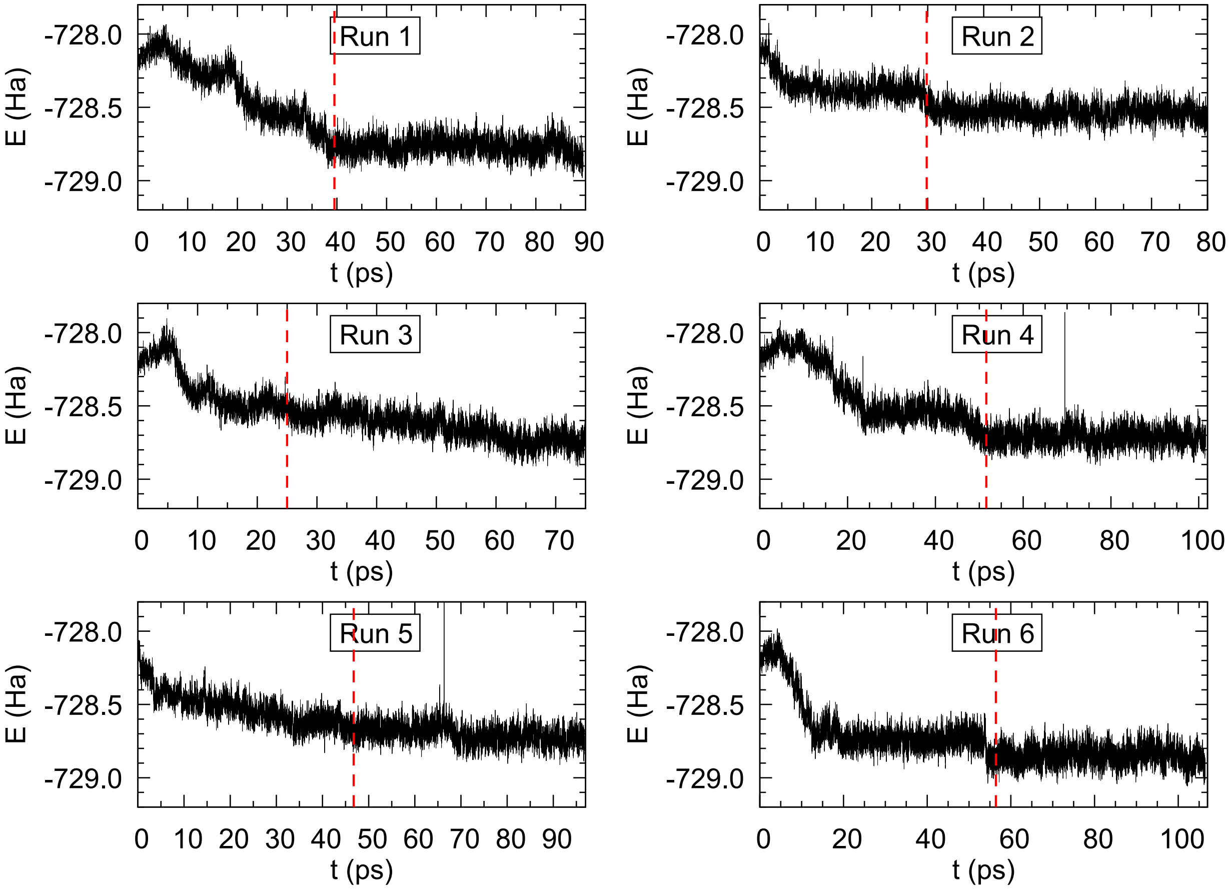

FIG. S1. Energy as a function of time in 6 NVT AIMD simulations of 32 CO + 32 H_2 at 1400 K, 13 GPa. The vertical dotted red line marks the time when the simulation was considered equilibrated.

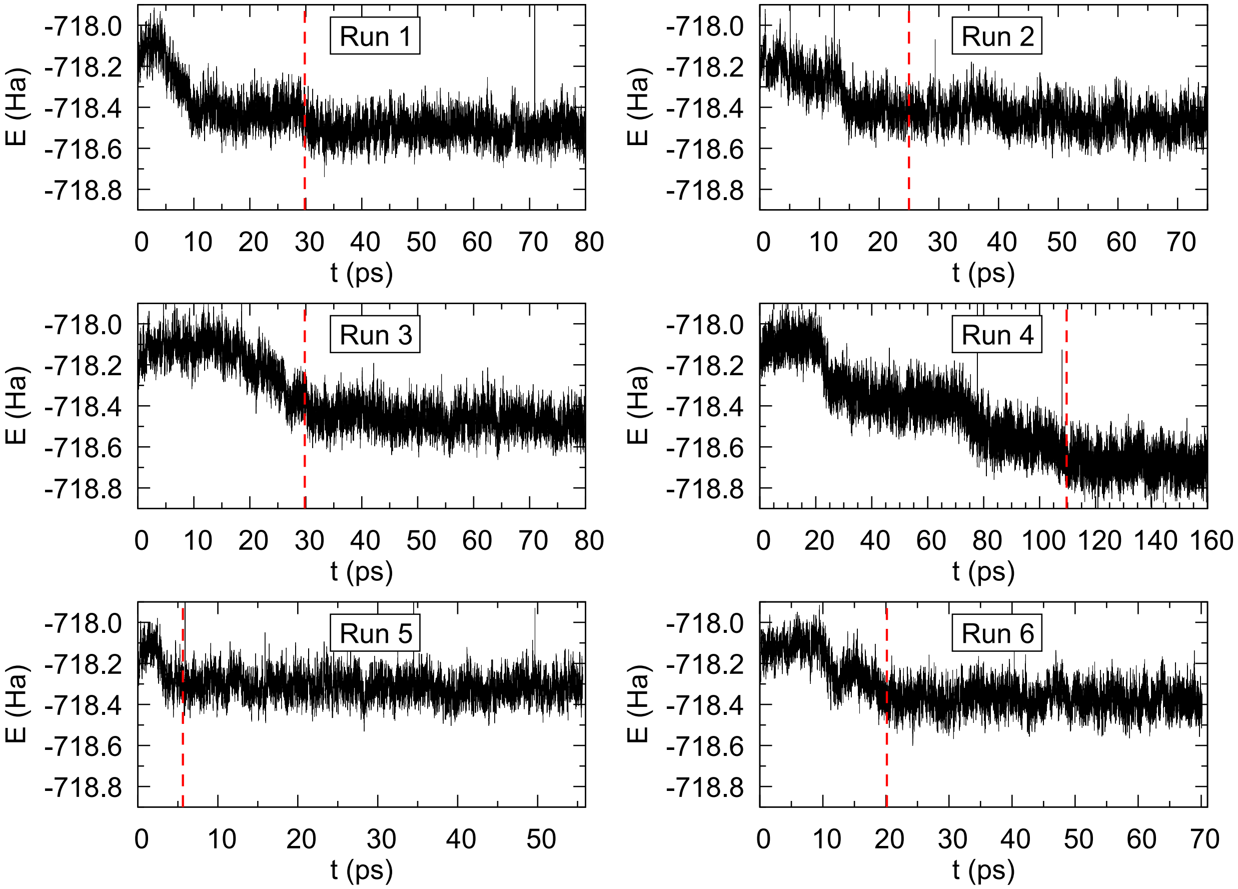

FIG. S2. Energy as a function of time in 6 NVT AIMD simulations of 18 CO + 18 H_2 + 18 H_2O at 1400 K, 13 GPa. The vertical dotted red line marks the time when the simulation was considered equilibrated.

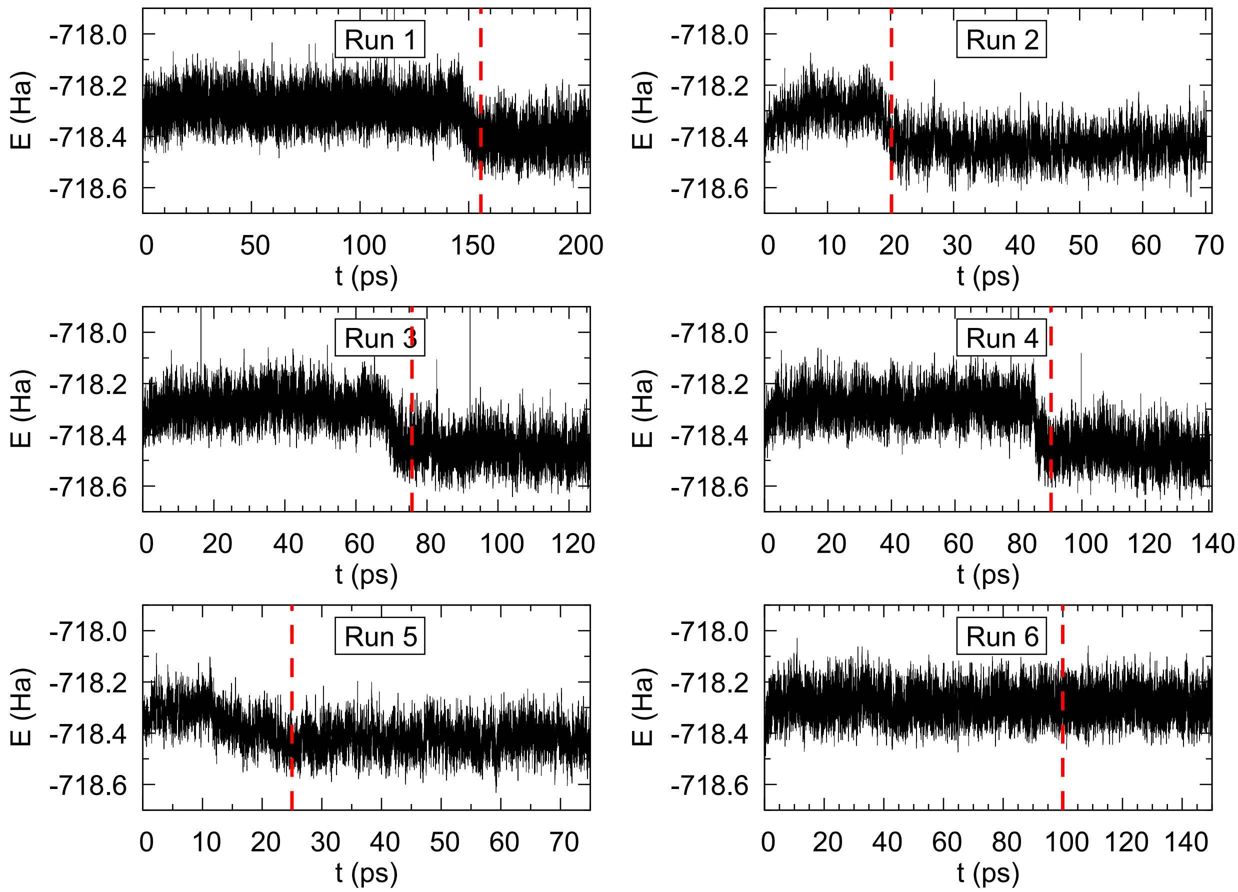

FIG. S3. Energy as a function of time in 6 NVT AIMD simulations of 18 CO + 18 H_2 + 18 H_2O at 1400 K, 10 GPa. The vertical dotted red line marks the time when the simulation was considered equilibrated.

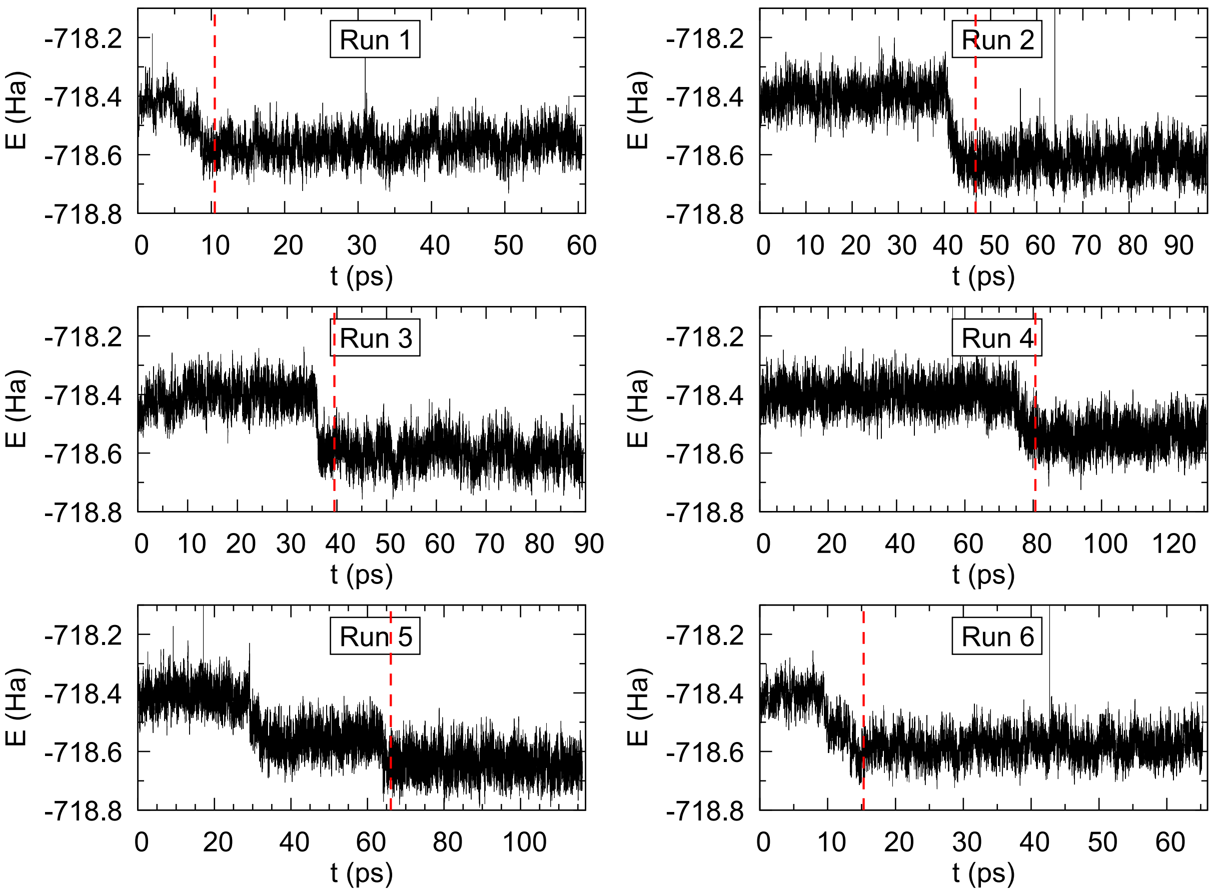

FIG. S4. Energy as a function of time in 6 NVT AIMD simulations of 18 CO + 18 H_2 + 18 H_2O at 1000 K, 10 GPa. The vertical dotted red line marks the time when the simulation was considered equilibrated.

TABLE SII. The first minimum after the first peak in the C-C and C-O RDFs, used to define the covalent bond length.

Starting composition	Simulation	P-T conditions	C-C	C-O
32 CO + 32 H_2	NVT	1400 K, 13 GPa	1.88 Å	1.85 Å
18 CO + 18 H_2 + 18 H_2O	NVT	1400 K, 13 GPa	1.87 Å	1.83 Å
18 CO + 18 H_2 + 18 H_2O	NVT	1400 K, 10 GPa	1.84 Å	1.79 Å
18 CO + 18 H_2 + 18 H_2O	NVT	1000 K, 10 GPa	1.83 Å	1.83 Å
32 CO + 32 H_2	NPT	1400 K, 13 GPa	1.95 Å	1.92 Å
18 CO + 18 H_2 + 18 H_2O	NPT	1400 K, 13 GPa	2.01 Å	1.91 Å

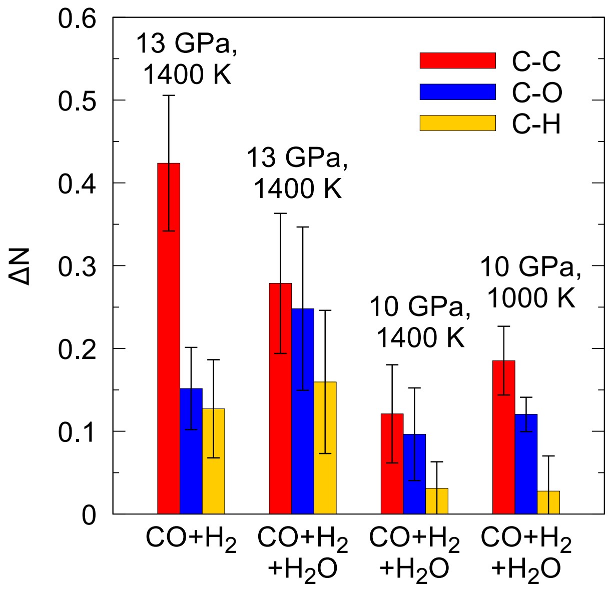

FIG. S5. ΔN, the change in the number of bonds per carbon atom. Error bars show standard deviations in the number of bonds per atom.

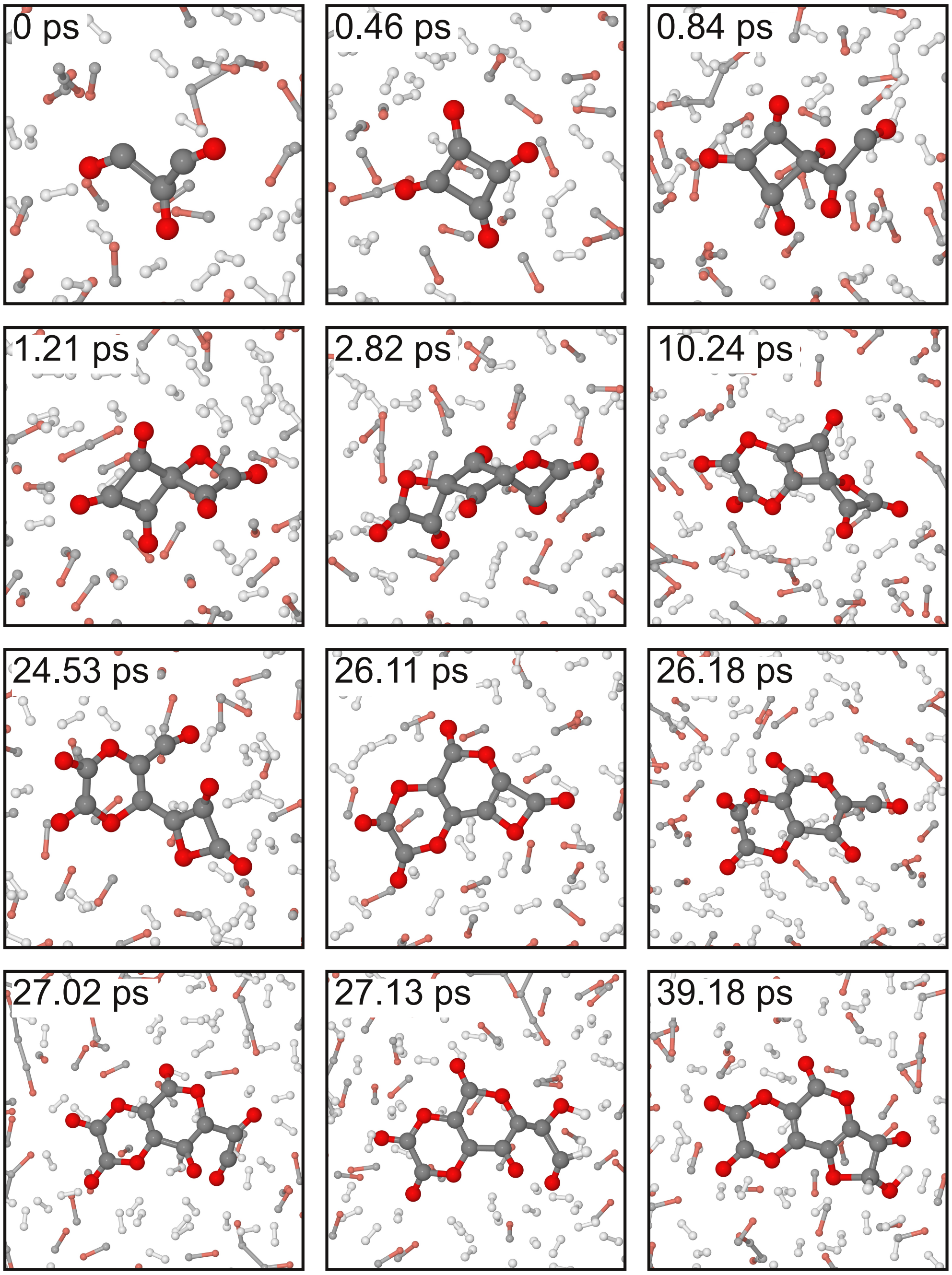

FIG. S6. Formation of a C_9 molecule with 5- and 6-membered rings from CO and H_2 at 13 GPa, 1400 K. There is no water in the solution.

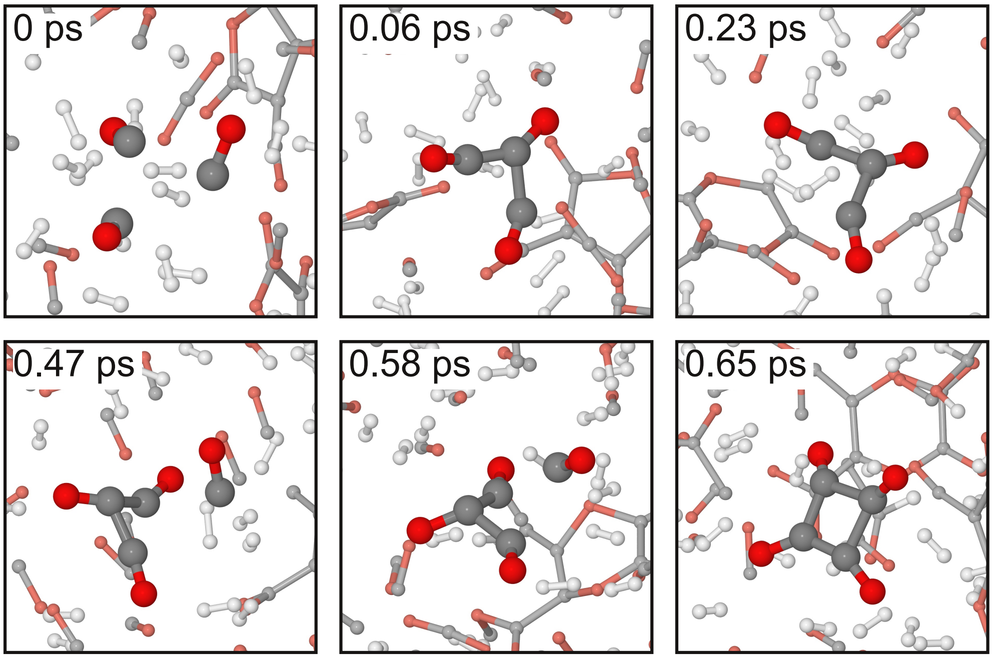

FIG. S7. Formation of cyclobutanetetrone from CO at 1400 K, 13 GPa. There is no water in the solution.

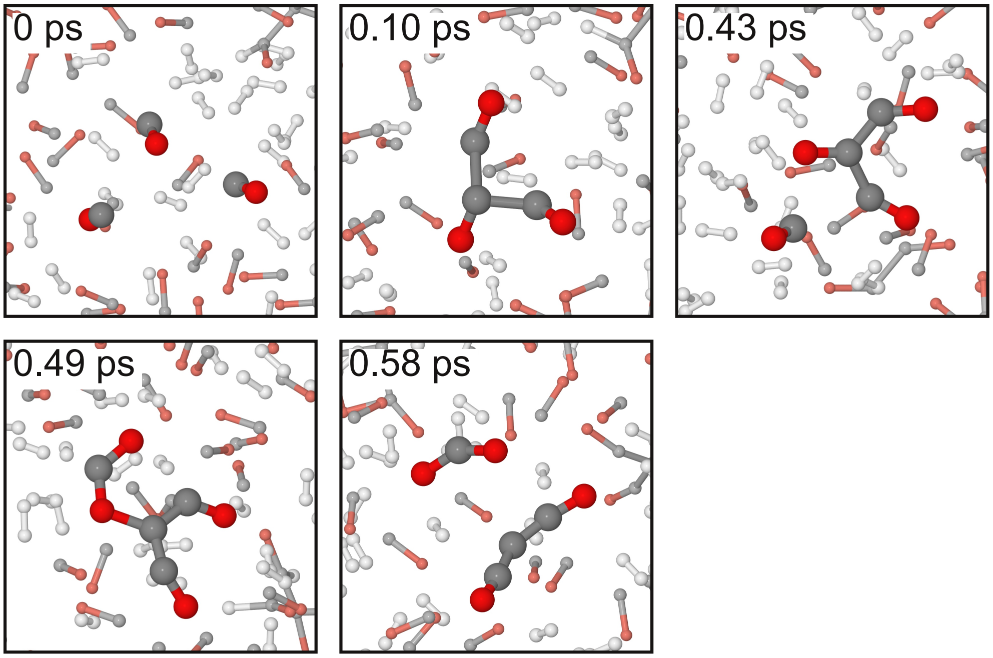

FIG. S8. Formation of carbon suboxide and CO_2 from CO at 1400 K, 13 GPa. There is no water in the solution.

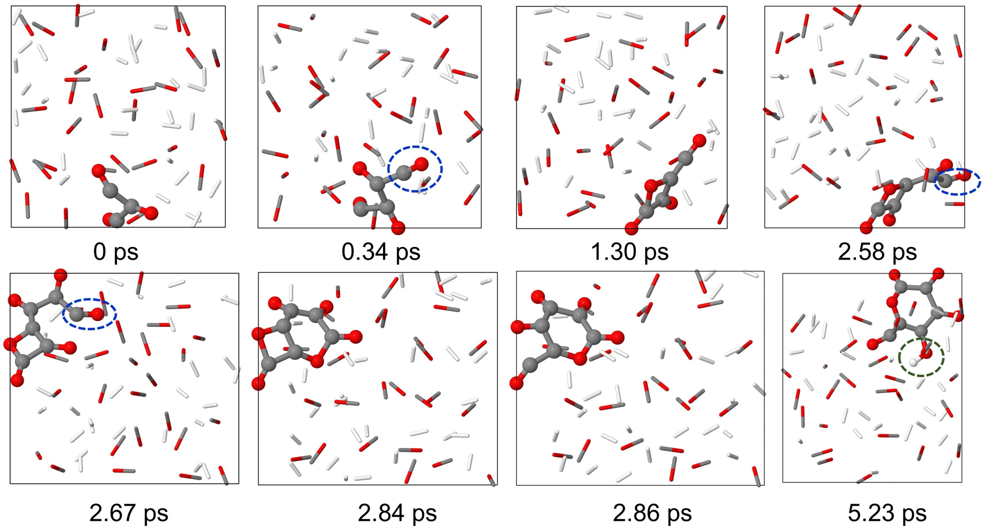

FIG. S9. The C_3 molecule formed in the solution without water reacted with CO at 0.34 ps, 2.58 ps, and 2.67 ps, producing C_4, C_5, and C_6 molecules, respectively. Subsequently, the C_6 molecule underwent cyclization and ring-opening processes, ultimately reacting with H_2 in the solution at approximately 5.23 ps to form a C–H–O compound. The pressure is 13 GPa and the temperature is 1400 K.

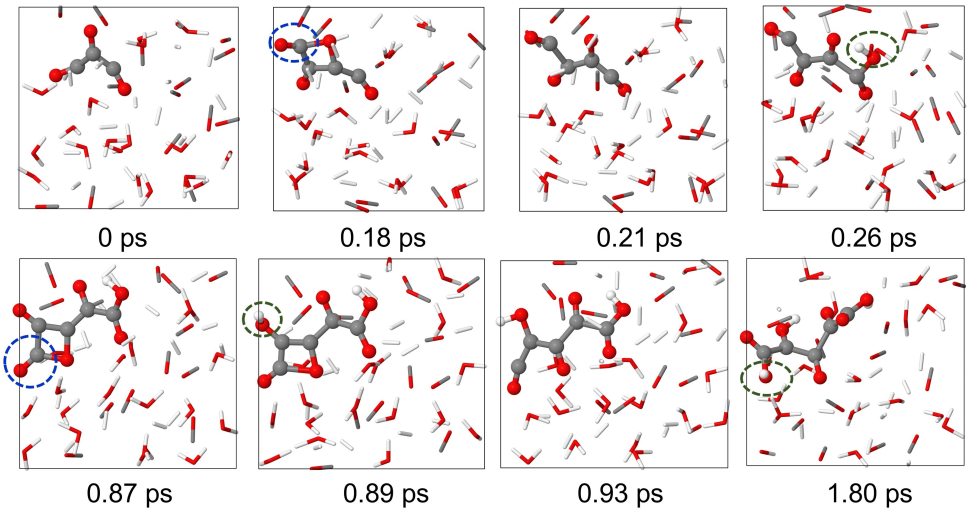

FIG. S10. The reaction initiated from a C_3 molecule, which reacted with CO at 0.18 ps to form a C_4 species and subsequently became a four-membered ring structure. At 0.26 ps, this C_4 intermediate reacted with hydrogen atoms from water to produce a C–H–O compound. Subsequently, this compound further interacted with CO and protons present in the aqueous solution, ultimately generating a more complex C–H–O compound. The pressure is 13 GPa and the temperature is 1400 K.
